# Outcomes in Lower Pole Kidney Stone Management Using Mini-Percutaneous Nephrolithotomy Compared With Retrograde Intra Renal Surgery: A Randomized Controlled Trial

**DOI:** 10.7759/cureus.35343

**Published:** 2023-02-23

**Authors:** Obaid Ur Rehman, Momal Imran, Mudessar Rafaqat, Fayyaz Ur Rahman Haider, Aveena Rehman, Umer Farooq, Shabbar H Changazi, Fazal Ur Rehman

**Affiliations:** 1 Urology, Ganga Ram Hospital, Lahore, PAK; 2 Community Medicine, Akhtar Saeed Medical and Dental College, Lahore, PAK; 3 Department of Urology, Services Hospital Lahore, Lahore, PAK; 4 Surgery/General Medicine/Trauma & Orthopedics/Neurology, Pakistan Institute of Medical Sciences, Islamabad, PAK; 5 Accident and Emergency, Provincial Headquarter Hospital Gilgit, Gilgit, PAK; 6 Department of General Surgery, Services Hospital Lahore, Lahore, PAK; 7 Department of Urology, Regional Headquater Hospital Gilgit, Gilgit, PAK; 8 Department of Urology, Shaikh Zayed Hospital Lahore, Lahore, PAK

**Keywords:** postoperative complications, stone clearance, retrograde intrarenal surgery, mini-pcnl, lower pole stone

## Abstract

Background

Because of the anatomical properties of the inferior calyx, lower pole stones are difficult to remove through the ureter, even if the stones are fragmented. Retrograde intra-renal surgery (RIRS) is typically employed to treat the smaller lower pole stones (1.0-2.0 cm) while percutaneous nephrolithotomy (PCNL) is primarily used to treat the larger diameter stones or when RIRS has failed to clear the stones. This study was conducted to compare mini-PCNL and RIRS for the management of lower pole kidney stones in terms of stone clearance.

Material and methods

This randomized control trial was conducted in the Department of Urology, Shaikh Zayed Hospital, Lahore from October 2020 to December 2022. A total of 150 patients between the ages of 18 and 80 years with a kidney stone size of 10-20 mm at the lower pole were included. Patients with positive urine culture, anatomical abnormalities, uncontrolled diabetes (hemoglobin{Hb}A1c >9%), and undergone previous renal surgery were excluded. Group A patients were treated with mini-PCNL, while group B patients were managed with RIRS. Follow-up visits were planned four weeks postoperatively with CT KUB (computed tomography of kidneys, ureters, and bladder) plain to assess stone clearance.

Results

The mean age in group A was 43.27 ± 13.86 years, while in group B was 45.32 ± 14.14 years. Out of 150 patients, 102 (68.0%) were males and 48 (32.0%) were females. Mean size of the stone was 15.30 ± 2.21 mm. Stone clearance after mini-PCNL was found in 69 (92.0%) patients and after RIRS in 59 (78.67%) patients (p-value = 0.021). Mean hospital stay after RIRS was 1.1 ± 0.09 days, while it was 2.3 ± 0.64 days after mini-PCNL (p-value < 0.001). Two (2.67%) patients in the mini-PCNL group developed bleeding postoperatively. The stone clearance rate in older patients (51 to 80 years) was significantly higher in the mini-PCNL group than RIRS group. Similarly, the stone clearance rate in female patients and in patients with larger stones (16 to 20 mm) was found to be higher in mini-PCNL group as compared to the RIRS group.

Conclusion

This study concluded that both mini-PCNL and RIRS are safe and efficient techniques for treating lower pole kidney stones with a size of 11-15 mm. However, mini-PCNL has a higher stone clearance rate compared to RIRS in the treatment of stones larger than 15 mm in size. This study further suggested that patients treated with mini-PCNL had a longer hospital stay compared to patients treated with RIRS.

## Introduction

Kidney stones are among the most common diseases in urology. It has a lifetime prevalence of 10% around the world. Males are more at risk of urolithiasis than females, with a ratio of 1.5:1. Kidney stones usually develop in saturated urine, which in turn depends on solute concentration, urine pH, ionic gradient, and complexation. Urinary stones are classified into five predominant types: calcium oxalate stones, calcium phosphate stones, struvite stones, uric acid stones, and finally cystine stones. Stones are composed primarily of calcium oxalate or phosphate (85%). Depending on the calcium content, kidney stones can be radiopaque or radiolucent. Calcium oxaltes, or phosphate stones, are radiopaque due to the increased amount of calcium in the stones. Uric acid and cystine stones are usually radiolucent due to decreased calcium composition. The clinical presentation includes acute, incessant colicky lumbar to groin pain, occurring in 50% of cases requiring intervention. In addition, 50% of affected patients will develop a relapse in their lifetime. The non-contrast CT scan is the most accurate imaging modality in diagnosing kidney stones [1,2]. 

Kidney stones usually arise in the lower pole of the kidney and account for 35% of all kidney stones. Treatment of lower pole kidney stones (LPS) depends on the composition of the stone, anatomical location, size, patient preference, clinical expertise, and equipment availability. Different treatment modalities for LPS range from less invasive extracorporeal lithotripsy (ESWL) to minimally invasive options with higher stone-free rates such as retrograde intrarenal surgery (RIRS) and percutaneous nephrolithotomy (PCNL) [3].

Retrograde intrarenal surgery (RIRS) is a widely used treatment option for a variety of urological disorders. Small kidney stones, calyx stones, blind calyces, and urothelial tumors of the upper urinary tract can be treated by RIRS using flexible ureteroscopes and lasers. Since it was reported as a treatment option for stone disease in 2002, the major limiting factor for RIRS has been larger stone size. Recently, some centers and surgeons have advocated RIRS to treat large stones with fewer complications and improved morbidity. The European Association of Urology (EAU) also recommended RIRS as an effective treatment for stones that are relatively larger in their recent guidelines [4]. PCNL is highly recommended for the treatment of large stone loads because of its higher success rate. However, it has higher complication rates, up to 25%, reported in some studies. With recent advances in technology and techniques, this equation has improved. Miniaturized PCNL (mini-PCNL) by using smaller instruments (nephroscopes) clears kidney stones with a high stone clearance rate and fewer complications [5,6].

There are very few studies comparing mini-PCNL and RIRS for the treatment of lower pole kidney stones. Therefore, this study was conducted to evaluate the comparison of RIRS and mini-PCNL in kidney stones with lower pole stones ranging in size from 10 mm to 20 mm.

## Materials and methods

This randomized controlled trial was conducted in the Department of Urology, Shaikh Zayed Hospital, Lahore from October 2020 to December 2022. Approval was obtained from the Hospital Ethical Committee Institutional Review Board and the Higher Board of Studies Shaikh Zayed Hospital before commencing the recruitment (approval number IRB/SZH/20/45). After taking informed consent, patients satisfying the inclusion criteria were recruited through the outpatient and emergency departments.

A sample size of 150 patients was computed with expected percentage of stone-free rate of 85% in patients treated with mini-PCNL and 97% in patients operated with RIRS by taking a confidence level of 5% and power of 80% [7]. Patients between the ages of 18 and 80 years of both sexes with kidney stones at the lower pole of size 10 mm to 20 mm as evaluated by CT KUB (computed tomography of kidneys, ureters, and bladder) plain were included in this study. Kidney stone patients with positive urine culture, anatomical anomalies determined by ultrasonography, uncontrolled diabetes (hemoglobin{Hb}A1c >9%), and undergone previous renal surgery were excluded from the study.

Patients were randomly segregated into groups A (mini-PCNL) and B (RIRS) using a random number table. Procedures were carried out under general anesthesia by consultant urologists who had at least three years of experience in the procedure. In group A (mini-PCNL) following procedure was done. A 6 Fr ureteral catheter was inserted into the tract using a cystoscope (KARL STORZ SE & Co., Tuttlingen, Germany), and a dye was instilled to opacify the pelvicalyceal system. After elaborating on the calyceal system through fluoroscopy, the affected calyceal was punctured and a 16 F sheath was used to dilate the tract. Mini-nephroscope 14 Fr was then introduced and stones were fragmented by holmium: YAG laser (Olympus, Beijing, China). Afterwards the collecting system was examined through a nephroscope (KARL STORZ SE & Co., Tuttlingen, Germany) and fluoroscopic confirmation was done to ensure stone fragmentation and clearance. In all cases, a 6 Fr 24 cm DJ stent (Bostone scientific, Marlborough, England) was inserted and the patient was discharged on postoperative day 2 with oral antibiotics.

In group B, RIRS was performed. In this procedure, a double J stent was placed to dilate the calyceal system two weeks prior to the surgery. During the procedure, a cystoscopy was performed, and a 0.035-inch guide wire was in the pelvi-calyceal system. A ureteric access sheath of 12 Fr was then positioned and with the help of a digital polyscope (Polydiagnost Gmbh, Hallbergmoos, Germany), the stone was fragmented using Holmium: YAG laser. DJ stent 6F 24 cm was placed in all the cases. In uneventful surgery, the patient was discharged on postoperative day 1 with oral antibiotics. Follow-up visits were planned four weeks postoperatively with CT KUB plain to assess stone clearance. Stone clearance was defined as no residual stone material found on the CT KUB plain in the lower pole of the kidney. Data was collected through a well-designed proforma.

SPSS version 23.0 (IBM Corp., Armonk, NY) was used for data analysis. Frequencies and percentages were computed for qualitative variables such as gender and stone clearance. Values were presented as mean ± standard deviation for quantitative variables such as age and hospital stay. Chi square test was used to evaluate categorical variables (stone clearance) and the t-test was done to compare continuous variables (hospital stay). Data was stratified for age, gender, and stone size to deal with effect modifiers. A value of p ≤0.05 was considered significant.

## Results

In this study, the mean age was 44.65 ± 14.09 years. The mean age in group A was 43.27 ± 13.86 years while in group B was 45.32 ± 14.14 years. Of 150 patients, 102 (68.0%) patients were male and 48 (32.0%) patients were female with a male to female ratio of 2.3:1. The mean stone size was 15.30 ± 2.21 mm (Table 1).

**Table 1:** Baseline characteristics of patients

		Group A	Group B	Total
Age (years)		43.27 ± 13.86	45.32 ± 14.14	44.65 ± 14.09
Gender	Male	52 (69.3% )	50 (66.7%)	102 (68%)
	Female	23 (30.7% )	25 (33.3% )	48 (32%)
Size of stones (mm)		15.29 ± 2.29	15.33 ± 2.18	15.30 ± 2.21

In this study, the stone clearance rate after mini-PCNL was found in 69 (92.0%) patients, and after RIRS stone clearance rate was found in 59 (78.67%) patients (p-value = 0.021). The mean hospital stay after RIRS was 1.1 ± 0.09 days while it was 2.3 ± 0.64 days after mini-PCNL (p-value < 0.001). Two (2.67%) patients in the mini-PCNL group developed bleeding postoperatively (Table 2).

**Table 2:** Comparison of outcomes

	Group A	Group B	p-value
Stone clearance	69 (92.0%)	59 (78.67%)	0.021
Hospital stay (days)	2.3 ± 0.64	1.1 ± 0.09	< 0.001
Complications	2 (2.67%)	0 (0%)	0.310

The stone clearance rate in elderly patients (51 to 80 years) was significantly higher in the mini-PCNL group than in the RIRS group. Similarly, it was found that the stone clearance rate was higher in the mini-PCNL group than in the RIRS group in female patients and patients with larger stones (16-20 mm) (Table 3).

**Table 3:** Stratification of stone clearance with respect to age and gender of the patient and size of stones

		Stone clearance in group A		Stone clearance in group B		p-value
		Yes	No	Yes	No	
Age (years)	18-50	49	06	50	06	0.974
	51-80	20	00	09	10	0.0001
Gender	Male	46	06	41	09	0.357
	Female	23	00	18	07	0.006
Size of stones (mm)	11-15	36	05	39	03	0.436
	16-20	33	01	20	13	0.0001

## Discussion

Because of the anatomical properties of the inferior calyx, lower pole stones are difficult to remove through the ureter, even if the stones are fragmented. RIRS is typically employed to treat the smaller lower pole stones (1.0-2.0 cm) while PCNL is primarily used to treat the larger diameter LPS or when RIRS has failed to clear the stones. With the increasing development of medical technology and technical expertise, the applicability of RIRS has increasingly extended to treat kidney stones larger than 2 cm or even larger than 3 cm [8]. On the other hand, many new PCNL technologies, including mini-PCNL and ultra-mini-PCNL (UMP), can not only treat larger kidney stones but also reduce the risk of kidney injury postoperatively. RIRS and UMP each have their own strengths and weaknesses, leading to controversy regarding the indication of these surgical procedures in the treatment of LPS. Compared to RIRS, PCNL can achieve a higher rate of stone fragmentation, although it carries greater surgical risk. A urologist named Janak Desai developed the ultra-mini percutaneous nephrolithotomy (UMP) in 2013 with a canal size of 11-14 F to lessen the risk of complications. As the percutaneous tract becomes smaller, the operation efficiency decreases and the intrarenal pressure may rise too high during the operation, causing damage to the kidney. Therefore, UMP is used to treat kidney stones smaller than 2 cm [9,10].

This study compared the min-PCNL and RIRS in terms of stone clearance rate, hospital stay, and postoperative complications. The results suggested that mini-PCNL had a better stone clearance rate as compared to RIRS however mini-PCNL was associated with longer hospital stay as compared to RIRS. Only two patients in the mini-PCNL group developed significant postoperatively bleeding requiring blood transfusion. Jiao et al. compared the results of mini-PCNL and RIRS in a meta-analysis of 8 randomized controlled trials (RCTs) in 725 patients with upper urinary calculi. They concluded that mini-PCNL had better efficacy in terms of stone clearance as compared to RIRS however mini-PCNL was associated with longer hospitalization time and a higher incidence of hematoma formation [11]. These results are in concordance with this study. Similarly another meta-analysis carried out by Gao et al. reported that mini-PCNL was more successful than RIRS for lower calyx stones in terms of stone clearance rate; however, RIRs involved shorter hospital stay and less hemoglobin fall [12]. Zheng et al. conducted a meta-analysis including two RCT and six controlled clinical trials and concluded that RIRS had similar results in terms of stone clearance as compared to mini-PCNL but RIRS was superior in terms of shorter hospital stay and lower complication rates [13]. Similar results were also quoted by Barone et al. in their meta-analysis [14] and Tsai et al. in their systemic review [15]. Although there is wide disparity in outcomes of mini-PCNL and RIRS in terms of stone removal, hospitalization, and complication rates for upper urinary tract kidney stones, given the existing literature, it is a generally accepted notion that RIRS is more effective in treating smaller stones (less than 2 cm) and PCNL is more effective on larger stones (more than 2 cm).

Of 75 patients in the mini-PCNL group, six patients had residual stones. Four of these had residual stones less than 4 mm in size and were treated conservatively, while two patients had stone sizes greater than 4 mm and were treated by extracorporeal shock lithotripsy. In the RIRS group, 16 patients had residual stones. Six patients had a stone size of less than 4 mm and were treated conservatively. Four patients opted for lithotripsy and the remaining patients underwent RIRS again. In the mini-PCNL group, two patients developed postoperative bleeding. Both patients were treated conservatively with blood transfusions and close monitoring.

This study has certain limitations. First, it is a single center study, which limits the generalization of our results to a broader population. Second, it has a smaller sample size. Multicenter studies with larger sample sizes will be beneficial in further evaluating the role of mini-PCNL and RIRS in the treatment of LPS.

## Conclusions

This study concluded that both mini-PCNL and RIRS are safe and efficient techniques for treating lower pole kidney stones with a size of 11-15 mm. However, mini-PCNL has a higher stone clearance rate compared to RIRS in the treatment of stones larger than 15 mm in size. This study further suggested that patients treated with mini-PCNL have a longer hospital stay compared to patients treated with RIRS. Therefore, the decision between the two procedures should be made based on anatomical parameters, particularly stone size, and patient preference.
